# Early Growth Response 1 (Egr1) Is a Transcriptional Activator of NOX4 in Oxidative Stress of Diabetic Kidney Disease

**DOI:** 10.1155/2018/3405695

**Published:** 2018-04-26

**Authors:** Fang Hu, Meng Xue, Yang Li, Yi-Jie Jia, Zong-Ji Zheng, Yan-Lin Yang, Mei-Ping Guan, Liao Sun, Yao-Ming Xue

**Affiliations:** ^1^Department of Endocrinology and Metabolism, Nanfang Hospital, Southern Medical University, Guangzhou, Guangdong, China; ^2^Department of Endocrinology and Metabolism, The Fifth Affiliated Hospital Sun Yat-Sen University, Zhuhai, Guangdong, China; ^3^Department of Endocrinology and Metabolism, Shenzhen People's Hospital, Second Affiliated Hospital of Jinan University, Shenzhen, Guangdong, China; ^4^Department of Geriatrics, Zhujiang Hospital, Southern Medical University, Guangzhou, Guangdong, China

## Abstract

**Background:**

NADPH oxidase 4 (NOX4) plays a major role in renal oxidative stress of diabetic kidney disease (DKD). NOX4 was significantly increased in Egr1-expressing fibroblasts, but the relationship between Egr1 and NOX4 in DKD is unclear.

**Methods:**

For the evaluation of the potential relationship between Egr1 and NOX4, both were detected in HFD/STZ-induced mice and HK-2 cells treated with TGF-*β*1. Then, changes in NOX4 expression were detected in HK-2 cells and mice with overexpression and knockdown of Egr1. The direct relationship between Egr1 and NOX4 was explored via chromatin immunoprecipitation (ChIP).

**Results:**

We found increased levels of Egr1, NOX4, and *α*-SMA in the kidney cortices of diabetic mice and in TGF-*β*1-treated HK-2 cells. Overexpression or silencing of Egr1 in HK-2 cells could upregulate or downregulate NOX4 and *α*-SMA. ChIP assays revealed that TGF-*β*1 induced Egr1 to bind to the NOX4 promoter. Finally, Egr1 overexpression or knockdown in diabetic mice could upregulate or downregulate the expression of NOX4 and ROS, and *α*-SMA was also changed.

**Conclusion:**

Our study provides strong evidence that Egr1 is a transcriptional activator of NOX4 in oxidative stress of DKD. Egr1 contributes to DKD by enhancing EMT, in part by targeting NOX4.

## 1. Introduction

Because of its high incidence and mortality rates, diabetic kidney disease (DKD) has become the most severe microvascular complication of diabetes mellitus [1]. In recent years, renal tubule fibrosis has been shown to crucially involved in the development of DKD [2]. Epithelial-mesenchymal transition (EMT), a characteristic of renal tubule fibrosis, eventually leads to renal fibrosis. During EMT, the epithelial cells lose their characteristic properties and gain mesenchymal features, resulting in upregulation of the mesenchymal protein alpha-smooth muscle actin (*α*-SMA) [3].

Genetic factors, abnormal glucose metabolism, and glomerular hemodynamic changes are also involved in DKD development. Among them, oxidative stress is a common mechanism [4]. NADPH oxidase 4 (NOX4), a member of the NADPH oxidases, has been confirmed to be the most important factor in DKD by generating superoxide and other reactive oxygen species (ROS) [5]. TGF-*β*1, a crucial pathogenic factor in DKD, can strongly induce NOX4 expression and is involved in the myofibroblast transformation [6]. Local activation of the renin-angiotensin system by angiotensin is regarded as the strongest factor contributing to NOX4 activation [7]. NOX4 is primarily expressed in renal tubule cells, and its upregulation indicates the degree of renal tubular injury in DKD [7]. Apocynin (Apo), a NOX inhibitor, has been shown to effectively alleviate kidney injury [8, 9].

Early growth response factor 1 (Egr1), a member of the immediate early response family, is highly expressed in a variety of kidney cells, including kidney proximal tubular epithelial cells [10]. Recent studies have indicated that Egr1 plays a crucial role in the pathogenesis of renal fibrosis by activating the promoters of collagen 1*α*1 (COL1A1), cartilage oligomeric matrix protein (COMP), periostin, matrix metalloproteinase 2 (MMP2) [11], tissue metallopeptidase inhibitor 1 (TIMP1), and osteopontin (OPN). Previous research in our department revealed that Egr1 has a crucial effect on the development of DKD by binding to the TGF-*β* promoter [12].

Using genome-wide expression profiling, Bhattacharyya [13] identified 647 genes whose expression was substantially changed by Egr1 in Egr1-expressing fibroblasts. One of these genes was NOX4, which showed an > eightfold increase. The increased expression of NOX4 caused by chronic hypoxia in pulmonary artery smooth muscle cells was mediated by Egr1 [14, 15]. In systemic sclerosis patients, increased expression of NOX4 enhances oxidative stress and further promotes fibrosis, during which elevation of Egr1 may play an important role [16, 17]. However, no studies have explored the direct relationship between Egr1 and NOX4. Through bioinformatics analyses, we discovered that the NOX4 gene contains putative Egr1-binding sites in its promoter region. To test this hypothesis, we investigated the regulation of NOX4 by Egr1 both in vitro and in vivo. The direct relationship between Egr1 and NOX4 was explored via chromatin immunoprecipitation (ChIP).

## 2. Materials and Methods

### 2.1. Animals

Male C57BL/6J mice (Animal Center of Guangdong province, 3-4 w) weighing 15-16 g received a high-fat diet (protein 26.2%, fat 34.9%, and carbohydrate 26.3%) for four weeks and then received a single injection of streptozotocin (STZ) (120 mg/kg, i.p., in citrate buffer, pH = 4.5, MP Biomedicals). Blood glucose was measured once a week, and a sustained blood glucose level of >16.7 mM for 16 weeks was considered an indicator of hyperglycemia. Control mice were injected with an equal volume of sodium citrate. The renal weight index was evaluated. At the end of 2 weeks of STZ injection, 30 mice were randomly assigned to one of five groups: HFD/STZ-induced diabetic mice (DM; *n* = 6), pcDNA3-Egr1-treated diabetic mice (EDM; *n* = 6), pcDNA3-vector-treated diabetic mice (PDM; *n* = 6), pGPU6-vector-treated diabetic mice (GDM; *n* = 6), and pGPU6-shEgr1-treated diabetic mice (shEDM; *n* = 6). EDM, PDM, GDM, and shEDM mice were administered the corresponding plasmid once a week for four weeks (12–16 w) via rapid injection of a large volume of DNA solution through the tail vein [18]. Animals were fed an HFD in a specific pathogen-free facility. Control and DM mice were sacrificed 12 weeks after modeling. EDM, PDM, GDM, and shEDM mice were sacrificed 16 weeks after modeling. Animal studies were conducted in accordance with the established institutional and state guidelines for the care and use of laboratory animals.

### 2.2. Metabolic Profile Analysis

At the end of the study, urine samples for 24 h were collected in metabolism cages. Blood samples were collected from the orbital sinus after inhalation of CO_2_ and fasting for 8 h. Triglycerides (TG), total cholesterol (TC), low-density lipoprotein (LDL) cholesterol, glucose, creatinine, and glycated hemoglobin (HbA1c) were detected using ELISAs (Shanghai Fanke Biotechnology Co., Ltd.) according to the manufacturer's instructions. Urinary albumin was determined by ELISA (Bethyl Laboratories Inc., Montgomery, TX, USA). Renal cortices were collected, quickly frozen in liquid nitrogen, and then stored at −80°C for later analysis.

### 2.3. Cell Culture and Transfection

Human proximal tubular epithelial (HK-2) cells were cultured as described previously [19]. Depending on the experiments, HK-2 cells were treated with recombinant human TGF-*β*1 (10 ng/mL; Gibco, New York, NY, USA), pENTER-Egr1 plasmid (2 *μ*g; Vigene Biosciences, Shandong, China), and small interfering RNA targeting Egr1 (siEgr1, 50 nM, RiboBio, Guangzhou, China). Transfections were performed using Lipofectamine™ 3000 reagent (Invitrogen, USA) following the manufacturer's instructions when cells were cultured to approximately 50–60% confluence in 12-well plates. Cells were harvested 48 h after transfection. TGF-*β*1 (10 ng/ml) was added to the culture medium 24 h before cell collection.

### 2.4. RNA Isolation and Real-Time Quantitative PCR (RT-qPCR)

Total RNA was extracted from renal cortices and HK2 cells with TRIzol (Dingguo, Beijing, China) according to the manufacturer's instructions. After reverse-transcription using *M-MLV* reverse transcriptase (Invitrogen, Carlsbad, CA, USA), the gene expression levels were determined by a Roche 480 thermal cycler using 40 ng of cDNA, SYBR Select Master Mix (Invitrogen), and the respective primers (Invitrogen, Table 1). The cycling conditions were described previously [12]. The relative mRNA expression level of each gene was calculated by the comparative 2^−ΔΔCt^ method, with *β*-actin as the reference.

### 2.5. Western Blotting Assays

Total protein was extracted from renal cortices and HK-2 cells using RIPA Lysis Buffer (Beyotime Institute of Biotechnology, Shanghai, China). Equal amounts of protein (20–50 *μ*g) were electrophoresed in 10% SDS-PAGE gels (Bio-Rad Laboratories, Hercules, CA, USA) and then were transferred to polyvinylidene fluoride (PVDF) membranes (Merck Millipore, MA, USA). PVDF membranes were blocked with 5% skim milk in 0.1% Tris-buffered saline with Tween-20 for 1 h and then incubated overnight with a primary antibody against Egr1 (1 : 400 dilution; Santa Cruz Biotechnology), NOX4 (1 : 200 dilution; Santa Cruz Biotechnology), or *α*-SMA (1 : 800 dilution; Santa Cruz Biotechnology). Finally, PVDF membranes were incubated with peroxidase-conjugated secondary antibody (1 : 15,000 dilution; LI-COR Biosciences, NE, USA) for 1 h at room temperature. Fluorescence was obtained using an Odyssey infrared imaging system (LI-COR) and quantified by ImageJ software.

### 2.6. Enzyme-Linked Immunosorbent Assay (ELISA)

Total protein was extracted from renal tissues using normal saline and a grinding machine. The samples were centrifuged for 10 min at 3000 ×g at 2–8°C within 30 min of collection and stored at −20°C or −80°C. The expression levels of NOX4, *α*-SMA, and ROS were detected using an ELISA (Shanghai Fanke Biotechnology Co., Ltd.) according to the manufacturer's instructions.

### 2.7. Kidney Histology and Immunohistochemistry

Kidney tissue was first embedded in paraffin and then cut into 4 *μ*m thick sections. Sections were stained with Masson's trichrome by standard protocol. To observe location and expression of target protein in kidney tissue, the sections were dealt with a series of steps below: dewaxing, closing endogenous peroxidase by hydrogen peroxide, antigen repairing, normal serum closing, dropping the first antibody(Egr1-, NOX4-, and *α*-SMA-specific antibodies), dropping biotinylated secondary antibody, dropping triple antibody (SAB complex), dropping tris Anti (SAB complex), and staining by hematoxylin. The pathological sections were observed under light microscope. Five high magnification (400x), perspectives were randomly selected from each samples. Semiquantitative analysis was performed adopting Image Pro-plus 6.0 software.

### 2.8. Chromatin Immunoprecipitation (ChIP)

ChIP was carried out using a ChIP-IT® Express kit (Active Motif, Bedford, MA, USA) according to the protocols. Anti-Egr1 antibody (Santa Cruz Biotechnology) or control IgG was used to cross-link protein DNA as shown in Table 2 (Sangon Biotech, Shanghai, China). RT-qPCR was used to detect the enrichment effect. We calculated the fold enrichment using the slope of the standard curve.

### 2.9. Dual-Luciferase Reporter Gene Assay

NOX4 promoter (-2000-0) luciferase reporter plasmid was constructed adopting the GV238-REPORT vector (GENE, Shanghai, China). 293 T cells were seeded in 24-well plates and transfected with 0.5 *μ*g of GV238 plasmid, 0.2 *μ*g of *β*-gal plasmid, pENTER-Egr1 plasmid (0, 0.2, 0.4, 0.6, 0.8, and 1.0*μ* g), and pENTER-vector plasmid (1.0, 0.8, 0.6, 0.4, 0.2, and 0 *μ*g). Lipofectamine® 3000 (Invitrogen, CA, USA), OptiMEM (Gibco, CA, USA), and *β*-gal were transfected as a transfection control. Cells were harvested 48 h after transfection and analyzed adopting luciferase assay kits (Beyotime, China). All experiments were performed in triplicate.

### 2.10. Statistical Analysis

Values are expressed as the mean ± SD. Two-tailed Student's *t-*test was used for two independent sample comparisons. Statistical analysis was performed using SPSS 13.0 software (IBM, IL, USA). The statistically significant level was set at *P* < 0.05.

## 3. Results

### 3.1. In Vivo Correlation between EMT in DM and Elevated Expression of Egr1, NOX4, and ROS

**Figure 1:**
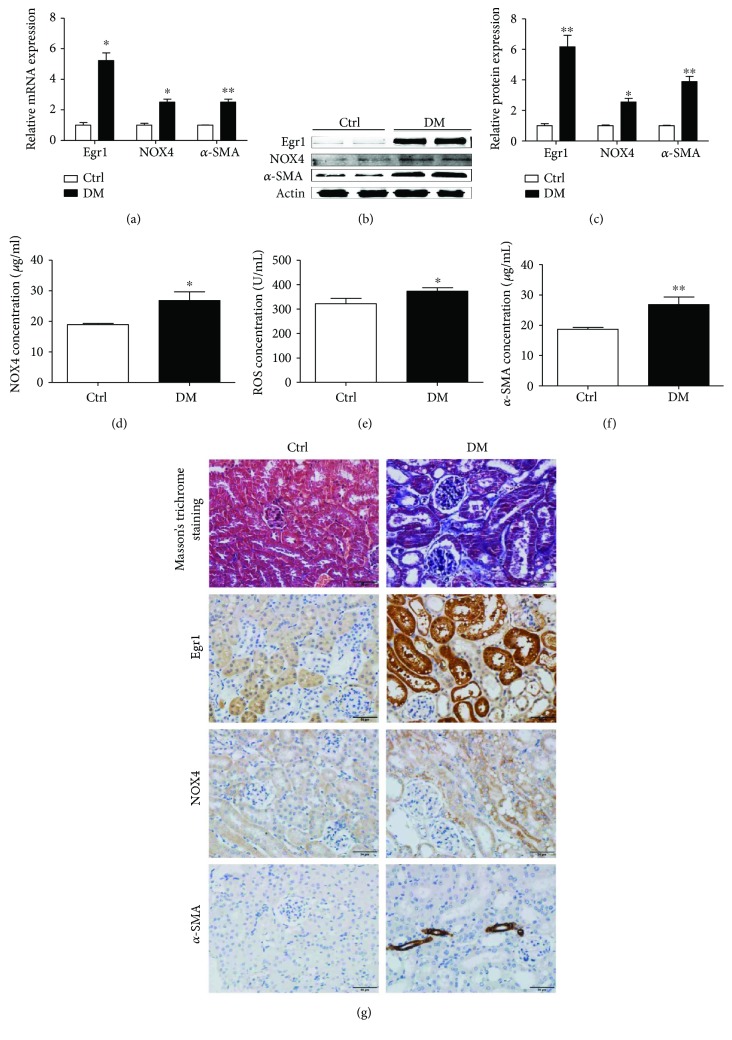
Egr1, NOX4, ROS, and *α*-SMA expression in control and HFD/STZ-induced diabetic mice at 12 weeks. (a) Levels of Egr1, NOX4, and *α*-SMA mRNA were measured using RT-qPCR. (b) Levels of Egr1, NOX4, and *α*-SMA protein were measured using Western blotting assays. (c) Semiquantitative levels of Egr1, NOX4, and *α*-SMA protein. (d) NOX4 concentration, (e) ROS concentration, and (f) *α*-SMA concentration were measured using ELISAs. The results are expressed as foldchange over baseline (control group). Values are the mean ± SD. ^∗^*P* < 0.05 and ^∗∗^*P* < 0.01 versus the control group. (g) Masson staining and Immunohistochemical staining of Egr1, NOX4, and *α*-SMA.

Based on the data shown in Table 3 and Masson's trichrome staining (Figure 1(g)), our animal model was successfully constructed. We examined renal cortices in mice at 12 weeks because a previous study showed that Egr1 was statistically higher in DM mice than in controls at this age [20].

Egr1 mRNA levels were fivefold higher in kidneys of DM mice than in kidneys of the control mice as shown by quantitative RT-PCR assays (Figure 1(a)). Egr1 protein levels were substantially higher in DM mice than in control mice as indicated by Western blotting assays (Figures 1(b) and 1(c)) and immunohistochemistry (Figure 1(g)). Furthermore, quantitative RT-PCR assays revealed that NOX4 and *α*-SMA expression was upregulated in the kidneys of DM mice compared to control mice (Figure 1(a)), which was further confirmed by Western blotting assays (Figures 1(b) and 1(c)), ELISAs (Figures 1(d) and 1(f)), and immunohistochemistry (Figure 1(g)). ROS, which are produced by NOX4, were shown to be increased in DM mice by ELISA (Figure 1(e)). In addition, NOX4 and *α*-SMA were mostly located in the renal tubule, while Egr1 was located in the glomerulus and renal tubule by immunohistochemistry (Figure 1(g)).

### 3.2. Changes in NOX4 Expression with Overexpression and Knockdown of Egr1 in TGF-*β*1-Treated HK-2 Cells

**Figure 2:**
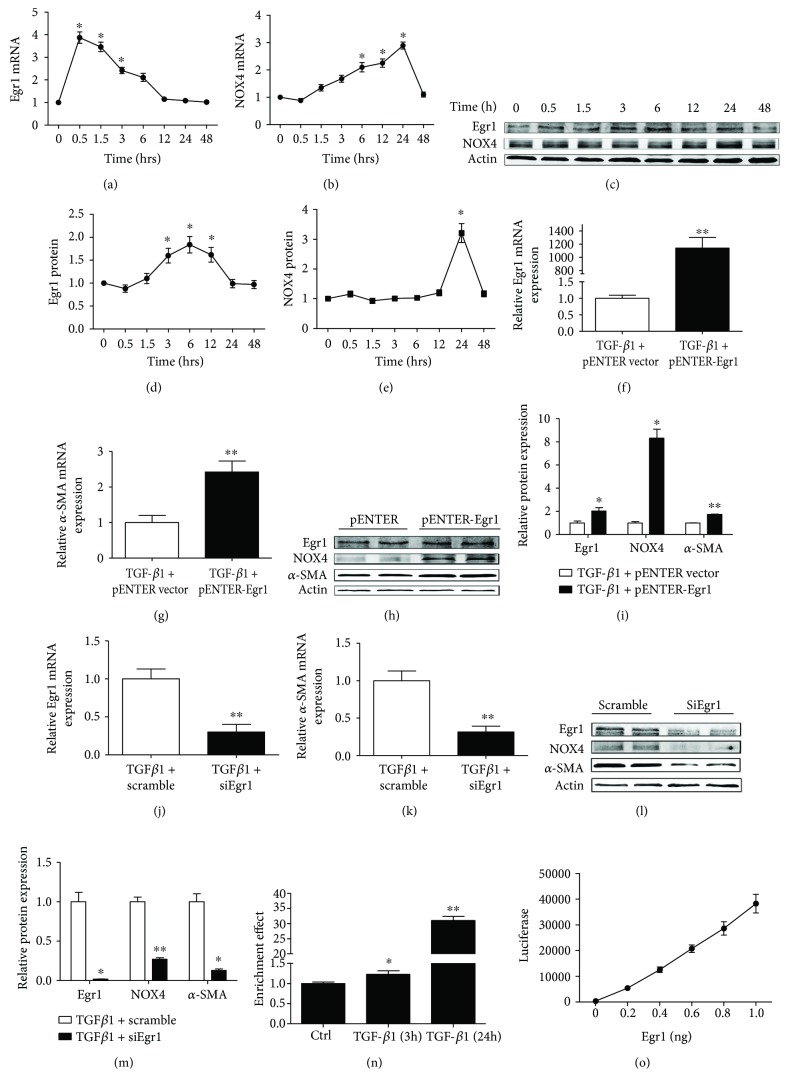
Changes in NOX4 expression with overexpression and knockdown of Egr1 in TGF-*β*1-treated HK-2 cells. (a, b, and c) Egr1 and NOX4 protein were measured using Western blotting assays in HK-2 cells treated with TGF-*β*1 (10 ng/mL) at 0 h, 0.5 h, 1.5 h, 3 h, 6 h, 12 h, 24 h, and 48 h. (d and e) Semiquantitative levels of Egr1 and NOX4 protein. (f) Cells were treated with either pENTER-Egr1 overexpression plasmid or with a pENTER vector for 48 h and then exposed for 3 h and 24 h to TGF-*β*1 (10 ng/mL). Levels of Egr1 were detected at 3 h, and levels of NOX4 and *α*-SMA were detected at 24 h after TGF-*β*1 (10 ng/mL). Levels of Egr1 mRNA were measured by RT-qPCR. (g) Levels of *α*-SMA mRNA were measured by RT-qPCR. (h and i) Levels of Egr1, NOX4, and *α*-SMA protein were measured using Western blotting assays. (j) Cells were either silenced with siEgr1 or treated with a scrambled control RNA for 48 h and then exposed for 24 h to TGF-*β*1 (10 ng/mL). Levels of Egr1 mRNA were measured using RT-qPCR. (k) Levels of *α*-SMA mRNA were measured using RT-qPCR. (l and m) Levels of Egr1, NOX4, and *α*-SMA protein were measured using Western blotting assays. The above results are expressed as foldchange over baseline. Values are the mean ± SD. ^∗^*P* < 0.05 and ^∗∗^*P* < 0.01 versus control group. (n) ChIP test to explore Egr1 binding to the NOX4 promoter. ChIP was performed using an anti-Egr1 antibody or a negative control IgG antibody in HK-2 cells treated for 3 h and 24 h with TGF-*β*1 (10 ng/mL). Immunoprecipitated DNA was subjected to RT-qPCR using specific NOX4 primers that included the Egr1 binding sites. (o) Dual-luciferase reporter gene assay to explore Egr1 binding to the NOX4 promoter.

To explore whether Egr1 and NOX4 further contribute to the DKD process, we tested Egr1 and NOX4 expression levels in TGF-*β*1-treated HK-2 cells. We chose HK-2 cells as the model because both Egr1 and NOX4 are present in these cells. TGF-*β*1-treated HK-2 cells are a classic cell model of DKD. We found increased Egr1 and NOX4 mRNA and protein levels under stimulation with recombinant TGF-*β*1 (10 ng/mL) (Figures 2(a)–2(e)).

Then, we explored the changes in NOX4 expression with overexpression and knockdown of Egr1 in TGF-*β*1-treated HK-2 cells. Transfection of cultured HK-2 cells with a plasmid encoding Egr1 (pcDNA3-Egr1) increased the expression of Egr1 (Figure 2(f)). The NOX4 protein expression was increased by eightfold (Figure 2(h) and 2(i)), and *α*-SMA was subsequently increased (Figures 2(g)–2(i)). In addition, transfection of cultured HK-2 cells with siEgr1 decreased the expression of Egr1 (Figure 2(j)). Subsequently, the NOX4 and *α*-SMA protein levels decreased (Figure 2 L-M). Next, HK-2 cells were stimulated with TGF-*β*1 (10 ng/mL) for 3 h and 24 h, and a ChIP assay was performed. TGF-*β*1 strongly induced Egr1 to bind to the NOX4 promoter over time. No enrichment was observed in the control IgG group (Figure 2(n)). The same results could be obtained by dual-luciferase reporter gene assay (Figure 2(o)).

### 3.3. Changes in NOX4 Expression with Overexpression and Knockdown of Egr1 in DM Mice

**Figure 3:**
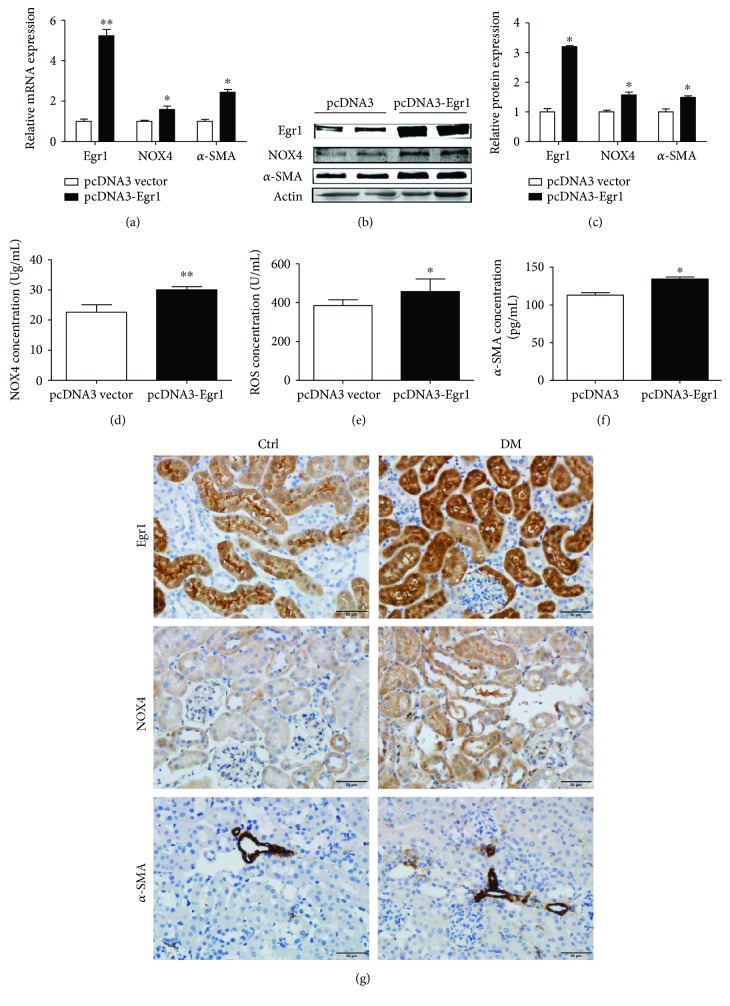
Egr1, NOX4, and *α*-SMA expression in HFD/STZ-induced diabetic mice transfected with pcDNA3-Egr1 plasmid. Diabetic mice were treated with either pcDNA3-Egr1 overexpression plasmid or with a pcDNA3 vector once a week for four weeks (12–16 w) via rapid injection of a large volume of DNA solution through the tail vein. (a) Levels of Egr1, NOX4, and *α*-SMA mRNA were measured using RT-qPCR. (b and c) Levels of Egr1, NOX4, and *α*-SMA protein were measured using Western blotting assays. (d) NOX4 concentration, (e) ROS concentration, and (f) *α*-SMA concentration were measured using ELISAs. The results are expressed as foldchange over baseline (pcDNA3 vector group). Values are mean ± SD. ^∗^*P* < 0.05 and ^∗∗^*P* < 0.01 versus the pcDNA3 vector group. (g) Immunohistochemical staining of Egr1, NOX4, and *α*-SMA.

**Figure 4:**
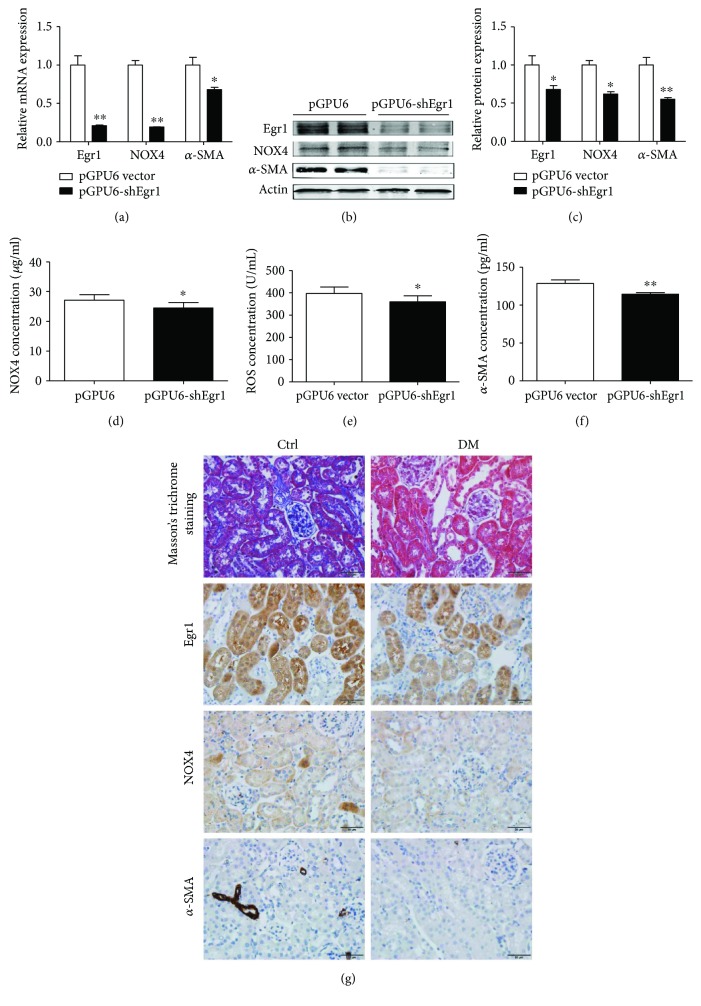
Egr1, NOX4, and *α*-SMA expression in HFD/STZ-induced diabetic mice transfected with pGPU6-shEgr1 plasmid. Diabetic mice were treated with either pGPU6-shEgr1 silencing plasmid or with a pGPU6 vector once a week for four weeks via rapid injection of a large volume of DNA solution through the tail vein. (a) Levels of Egr1, NOX4, and *α*-SMA mRNA were measured using RT-qPCR. (b and c) Levels of Egr1, NOX4, and *α*-SMA protein were measured using Western blotting assays. (d) NOX4 concentration, (e) ROS concentration, and (f) *α*-SMA concentration were measured using ELISAs. The results are expressed as foldchange over baseline (pGPU6 vector group). Values are mean ± SD. ^∗^*P* < 0.05 and ^∗∗^*P* < 0.01 versus the pGPU6 vector group. (g) Masson staining and immunohistochemical staining of Egr1, NOX4, and *α*-SMA.

Hydromechanics, which has been effectively used for transfecting mice with plasmids for thirty years [21], was used to induce Egr1 overexpression or knockdown in DM mice in this study. Egr1 mRNA and protein expression increased 5.2-fold and 3.3-fold in the EDM group compared with the vector group, respectively (Figures 3(a)–3(c) and 3(g)). Subsequently, the NOX4 and *α*-SMA mRNA and protein levels increased (Figures 3(a)–3(c) and 3(g)). The NOX4, ROS, and *α*-SMA protein levels measured by ELISAs also showed the same results (Figures 3(d)–3(f)). In addition, Egr1 levels were decreased in the SEDM group (Figure 4(a)). NOX4 was subsequently significantly reduced (Figure 4(a)). *α*-SMA, the molecular signature of renal myofibroblasts, was also decreased (Figure 4(a)). Similar results were obtained using ELISAs, Western blotting, and immunohistochemistry (Figures 4(b)–4(g)).

## 4. Discussion

DKD is a serious complication of diabetes mellitus. Currently, there is no effective treatment for DKD [22, 23]. Thus, there is an urgent need to identify novel targets. Oxidative stress is considered the most common pathway promoting kidney injury in DKD [24, 25]. The glucose-TGF-*β*1-NOX4-ROS pathway has been accepted worldwide. In the present study, we further extended the previous conclusion and first demonstrated that Egr1 can bind to the NOX4 promoter and is involved in the development of DKD. Importantly, we demonstrated that Egr1 knockdown mice showed alleviation of EMT due to downregulation of NOX4.

Egr1 expression is low or undetectable in resting cells. However, its expression can be elevated rapidly and transiently following exposure to various extracellular stimuli. Previous work from our department has revealed a change in Egr1 following high glucose and TGF-*β*1 stimulation in NRK-52E, HMC, and HK-2 cells [12, 19, 26]. This paper reports similar results. Egr1 is induced in a rapid and transient manner via the negative feedback of NGFI-A binding protein 2 (Nab 2). Egr1/Nab 1 and Nab 2 can be recognized by the inhibitory domain of Egr1 protein [27]. Then, cofactors recruit the inhibitory nucleosomal remodeling and deacetylation (NuRD) complex to promoter-bound Egr1 and ultimately repress Egr1-dependent transcriptional activity [28]. Egr1 is regarded as a fibrosis factor in kidney fibrosis. Ho et al. [29] found that Egr1 knockout mice exhibited alleviated fibrosis and inflammatory changes; in addition, primary HK-2 cells with knockout of Egr1 exhibited an attenuated reaction to TNF-*α* and TGF-*β*. However, there is little research regarding the contribution of Egr1 to fibrosis in DKD. Recent studies have shown increased Egr1 gene and protein expression in DM and rats [19, 20] but did not determine whether Egr1 promoted kidney fibrosis in DKD. Using Egr1 and shEgr1 plasmid-treated DM mice, we showed here that Egr1 can promote the development of DKD.

There have been many studies investigating the association between NOX4 and kidney injury. However, the conclusions in different models vary. Renal NOX4 has been suggested to be either protective or not involved in UUO and 5/6Nx models [19, 20], while it has been definitively shown that NOX4 can promote DKD development [30]. NOX4 exhibits very low constitutive activity, which can be highly upregulated in response to various stimuli, such as high glucose, TGF-*β*, and AngII [31, 32]. In the present study, both NOX4 mRNA and protein were overexpressed in the diabetic renal cortex and TGF-*β*1-treated HK-2 cells, indicating that NOX4 is fully activated in the diabetic kidney. These results suggest that NOX4 is involved in the development of DKD. Our findings are similar to those reported in a previous study.

Then, we explored the relationship between Egr1 and NOX4 in vivo and in vitro. NOX4 expression increased following treatment of HK-2 cells and diabetic kidney mice with the Egr1 plasmid. Conversely, NOX4 decreased following Egr1 knockdown in HK-2 cells and diabetic kidney mice. A direct relationship between Egr1 and NOX4 has not been previously reported. We are the first to show that Egr1 can directly bind to the NOX4 promoter to improve DKD. These results offer further evidence that Egr1 can promote kidney fibrosis in addition to binding to the promoters of COL1A1, COMP, periostin, MMP2, TIMP1, and OPN. Surprisingly, we found that knockdown of Egr1 in mice led to decreased *α*-SMA expression, a characteristic of EMT. Our research extends previous work by providing strong evidence that Egr1 contributes to DKD by promoting NOX4 expression. Therefore, we concluded that Egr1 knockdown can ameliorate DKD in part by decreasing NOX4. Thus, Egr1 may be a possible target for DKD treatment.

However, our study has some limits. Microalbuminuria, the inflammation index, and other factors in addition to *α*-SMA should be examined to explore and confirm the nephroprotective role of Egr1 in DKD. Additionally, an Egr1 inhibitor should be used to treat DM mice to obtain more convincing results. These experiments will be part of subsequent work performed in this department.

## 5. Conclusion

Our research demonstrates that Egr1 is a transcriptional activator of NOX4 in DKD. Egr1 contributes to DKD by enhancing EMT, in part by targeting NOX4.

## Figures and Tables

**Table 1:** Sequences of primers for quantitative RT-PCR used in this study.

Gene	Primers
hEgr1	(f) CTGACCGCAGAGTCTTTTCCTG (r) TGGGTGCCGCTGAGTAAATG
mEgr1	(f) CCTTTTCTGACATCGCTCTGAA (r) CGAGTCGTTTGGCTGGGATA
hNOX4	(f)CTTTTGGAAGTCCATTTGAG (r) CGGGAGGGTGGGTATCTAA
mNOX4	(f) ACAATCTTCTTGTTCTCCTGCT (r) CATCCTTTTACCTATGTGCCG
h*β*-actin	(f) CCCTGGACTTCGAGCAAGAGAT (r) GTTTTCTGCGCAAGTTAGG
m*β*-actin	(f) CGAGCGTGGCTACAGCTTCA (r) AGGAAGAGGATGCGGCAGTG
h*α*-SMA	(f) ATCCTCCCTTGAGAAGAGTT (r) ATGCTGTTGTAGGTGGTTTC
m*α*-SMA	(f) TGGATCAGCGCCTTCAGTTC (r) GGCCAGGGCTAGAAGGGTA

**Table 2:** Primer sequences for PCR of ChIP-enriched DNA used in this study.

Gene	Primers
hNOX4	(f) ATCTGGAGGCTCTGCTGGTA (r) GGCATGCTGTGAGAAGTTCA

**Table 3:** Metabolic Profile Analysis of mouse parameters.

	Ctrl	DM
Glucose (mM)	5.65 ± 0.34	22.38 ± 1.13^b^
HbA1c (%)	5.31 ± 0.40	9.83 ± 0.56^b^
Urine microalbumin (*μ*g/24 h)	6.23 ± 0.55	78.75 ± 4.06^b^
Renal weight index (mg/kg)	6.42 ± 0.45	15.57 ± 1.12^b^
Body weight (g)	27.31 ± 1.97	23.30 ± 1.46
Creatinine (mM)	177.67 ± 12.54	174.15 ± 2.31
CH (mM)	3.77 ± 0.24	6.12 ± 0.58^b^
TG (mM)	5.06 ± 0.40	7.54 ± 0.51^b^
LDL-C (mM)	2.12 ± 0.12	4.06 ± 0.23^b^

Mean ± SD; *n* = 6; ^a^*P* < 0.05 and ^b^*P* < 0.01 versus control
